# Liquefaction of renal allograft: An unprecedented complication in transplantation medicine

**DOI:** 10.1177/1742271X251337256

**Published:** 2025-05-06

**Authors:** Salman J Arain, Adam H Morrell

**Affiliations:** 1 Leeds Teaching Hospitals NHS Trust, Leeds, UK; 2 Faculty of Medicine and Health, University of Leeds, Leeds, UK

**Keywords:** Renal allograft failure, allograft liquefaction, transplant nephrectomy considerations, immunosuppression management, dialysis reinitiation timing, complications post-transplant, multidisciplinary approach, rare renal complications, unique transplant complications, individualised treatment strategies

## Abstract

**Background:**

Renal transplantation stands as the gold standard treatment for end-stage renal disease, offering improved quality of life and increased survival compared to dialysis. However, despite its advantages, renal allograft failure can occur, necessitating careful management to mitigate associated complications. Instances of liquefaction of the transplanted kidney are exceedingly rare, posing unique challenges to clinicians in the management of allograft failure. Existing literature highlights the complexities surrounding decisions regarding dialysis reinitiation, immunosuppression management, and the potential need for transplant nephrectomy in patients with failed renal allografts.

**Case presentation:**

A 52-year-old Caucasian female with a complex medical history including stage 5 chronic kidney disease, non-ST-segment elevation myocardial infarction, hypertrophic cardiomyopathy with mild left ventricular systolic dysfunction, atrial fibrillation, and a history of cardiac arrest resulting in hypoxic brain injury, presented with a unique complication following renal transplantation. Imaging studies revealed complete breakdown and liquefaction of the transplanted kidney in the right iliac fossa. Despite interventions such as ultrasound-guided aspiration and drainage, the patient’s condition continued to deteriorate.

**Conclusion:**

This case highlights a rare occurrence of allograft liquefaction following renal transplantation, emphasising the need for vigilance in monitoring transplant recipients for uncommon complications. The management of such cases requires a multidisciplinary approach, considering factors such as dialysis reinitiation timing, immunosuppression management, and the potential need for transplant nephrectomy. Further research is warranted to elucidate the pathophysiology and optimal management strategies for unique complications such as allograft liquefaction, underscoring the importance of individualised treatment approaches in complex clinical scenarios.

## Background

Renal transplantation stands as the gold standard treatment for end-stage renal disease (ESRD), offering improved quality of life and increased survival compared to dialysis. However, despite its advantages, renal allograft failure can occur, necessitating careful management to mitigate associated complications. While strategies such as dialysis reinitiation timing, immunosuppression management, and the consideration of transplant nephrectomy have been explored in the context of allograft failure, instances of liquefaction of the transplanted kidney are exceedingly rare.^1,2^

Existing literature highlights the challenges clinicians face when managing patients with failed renal allografts, particularly regarding the decision-making process concerning dialysis reinitiation, immunosuppression management, and the potential need for transplant nephrectomy.^3^ Immunosuppression withdrawal after allograft failure may trigger allograft intolerance syndrome, leading to manifestations akin to general infections. Allograft intolerance syndrome commonly develops within the first year after restarting dialysis, triggered by the withdrawal of immunosuppression.^3^ Symptoms resemble general infections, including fever, flu-like symptoms, haematuria, and increased allograft size or tenderness. Woodside et al.^4^ found that within 6 months of allograft failure, 44% of patients were hospitalised with fever, with documented infections in only 38% of those who stopped immunosuppression. Risk factors for allograft nephrectomy due to allograft intolerance syndrome include donor age, rejection history, and shorter allograft survival.^3^ Continuation of immunosuppression post-allograft failure presents its own set of challenges, with increased risks of infectious complications and adverse cardiovascular events.^3^ Continuing immunosuppressive therapy in allograft failure patients raises mortality and morbidity risks. A cohort study of 197 patients by Smak Gregoor et al. found those on low-dose maintenance immunosuppression post-allograft failure showed significantly higher rates of viral, bacterial, and opportunistic infections per patient-year (1.7 versus 0.51; P < 0.0001). Mortality rates linked to cardiovascular and infectious complications were also higher in patients continuing immunosuppression compared to those who stopped.^5^

## Case presentation

A 52-year-old Caucasian female with a complex medical history presented with a unique complication following renal transplantation. Comorbidities prior to renal transplant included stage 5 chronic kidney disease (CKD) due to reflux nephropathy necessitating haemodialysis since April 2011, hypertrophic cardiomyopathy with mild left ventricular systolic dysfunction (LVSD), atrial fibrillation (AF), an implantable cardioverter-defibrillator (ICD), and amiodarone-induced thyroid deficiency. Comorbidities following the renal transplant included a Hartmann’s procedure for perforated diverticulitis, cardiac arrest, and subsequent hypoxic brain injury, and NSTEMI with angioplasty.

The patient underwent renal transplantation in 2019. Prior to transplantation, the patient’s estimated glomerular filtration rate (eGFR) was approximately 12 ml/min. Post-transplant, renal function initially climbed to a peak of eGFR 43 ml/min (creatinine 116 umol/L). However, 3 weeks later, she began experiencing a decline in renal function to eGFR 18 ml/min (creatinine 254 umol/L). The function improved gradually without any intervention to eGFR 44 ml/min (creatinine 114 umol/L). Serial ultrasound scans at this point demonstrated normal appearances and Doppler evaluation. Four weeks later, she presented with worsening renal function, with a gradual decrease to a minimum of eGFR 16 ml/min (creatinine 280 umol/L) and abdominal pain localised to the transplant site. This coincided with her admission for perforated diverticulitis, for which she underwent a Hartmann’s procedure. A biopsy of the transplant kidney at this point showed acute cellular rejection with morphological features consistent with antibody-mediated rejection. This was treated with methylprednisolone and plasma exchange. For approximately 3 months, her renal function stabilised at around eGFR 20 ml/min. However, despite immunosuppression for the acute cellular rejection and antibody-mediated rejection, which was re-demonstrated on a second biopsy, her condition continued to deteriorate, culminating in recurrent admissions characterised by fever, abdominal pain, and vomiting. Her immunologic status revealed a low level of donor-specific antibodies (DSA) with a progressively decreasing eGFR to a nadir of 9 ml/min. Subsequently, she was commenced on haemodialysis approximately 9 months after her renal transplant due to a failed renal allograft.

**Figure 1.**
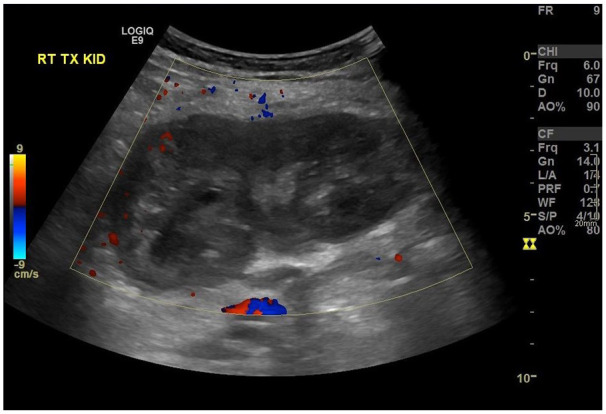
Initial ultrasound: Right iliac fossa transplant kidney, devoid of blood flow and oedematous.

**Figure 2.**
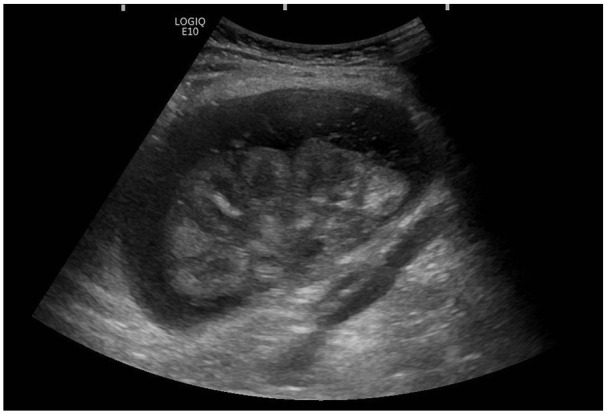
Ultrasound one month later: Shallow 2.5-cm fluid collection containing debris encasing the transplant kidney.

**Figure 3.**
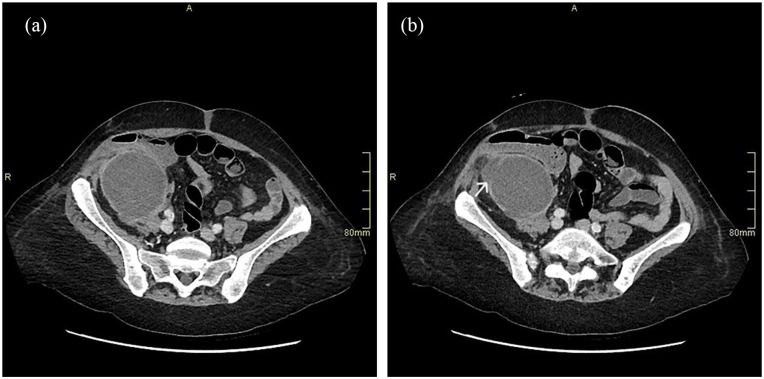
CT Abdomen demonstrating transplant kidney breakdown and liquefaction: (a) fluid collection increase and (b) wall degradation (arrow).

**Figure 4.**
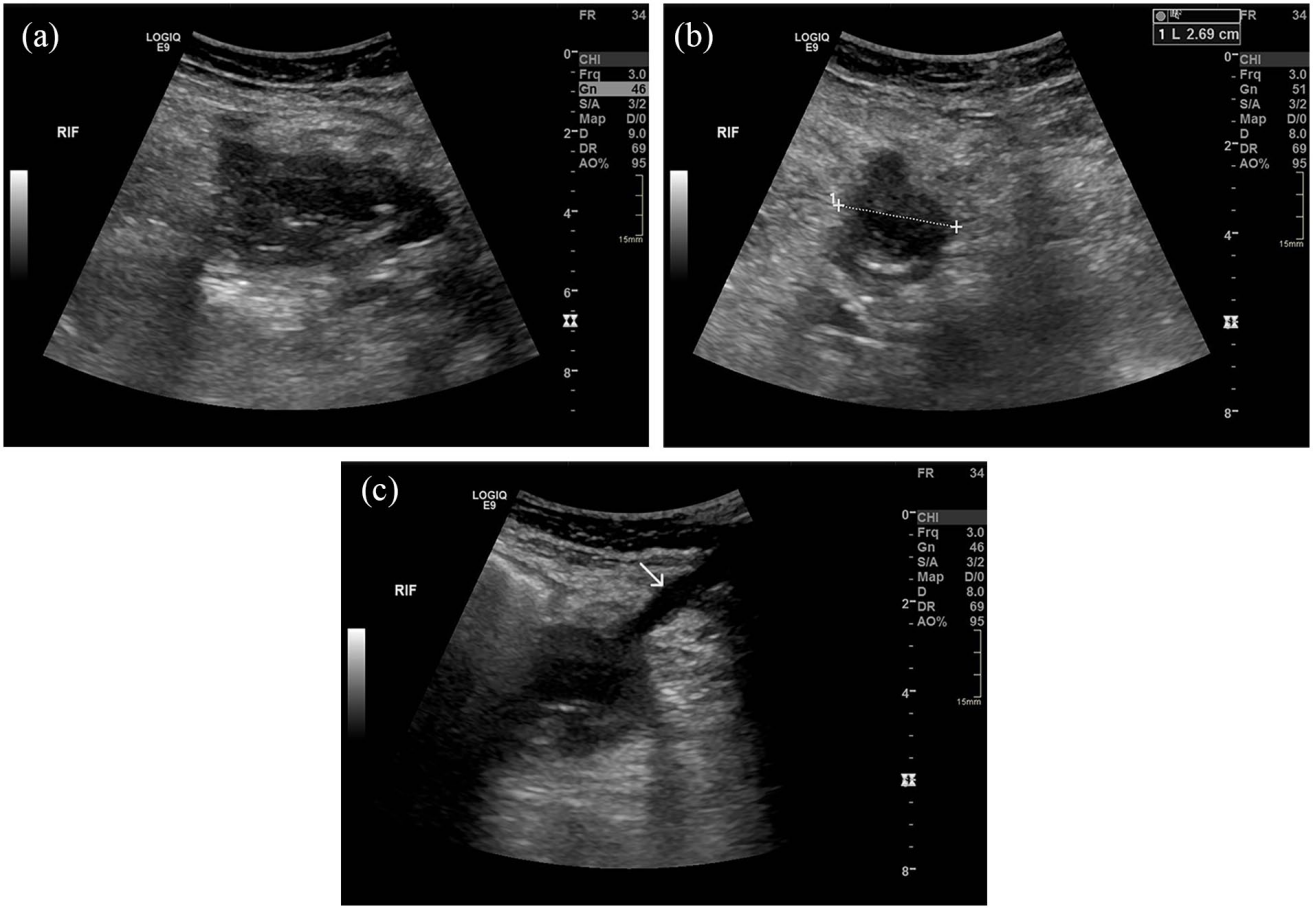
Post-drainage ultrasound: (a) long-section of a remnant complex fluid collection, (b) transverse view, and (c) drain track (arrow).

Post-transplant failure, the patient presented in 2021 with rising CRP (145 mg/L), borderline pyrexia (37.8°C), and tenderness over the allograft site. Ultrasound imaging revealed an avascular, oedematous kidney with evidence of infection, including urothelial thickening (Figure 1). Follow-up ultrasound imaging 4 weeks later demonstrated early stages of breakdown with the transplant encapsulated by a fluid collection containing debris (Figure 2). Subsequent computed tomography (CT) scans demonstrated complete breakdown and liquefaction of the transplanted kidney in the right iliac fossa (Figure 3). Efforts to manage the complication included ultrasound-guided aspiration and drainage procedures, with subsequent follow-up indicating a persistent but diminishing fluid collection encapsulating the parenchyma (Figure 4).

## Conclusion

This case represents a rare occurrence of liquefaction of a transplanted kidney following renal transplantation. Despite the extensive literature on allograft failure management, instances of complete breakdown and liquefaction of the allograft are scarcely documented. The management of such cases necessitates a multidisciplinary approach, considering factors such as the timing of dialysis reinitiation, immunosuppression management, and the potential need for transplant nephrectomy.

The management of failed kidney allografts is complex and individualised, as outlined in the literature. Failed allografts may result in systemic complications, including chronic inflammation, increased infection risks, and heightened cardiovascular mortality.^1,3,6^ Liquefaction further complicates this scenario by potentially introducing severe localised and systemic sequelae, necessitating urgent intervention. A retained failed allograft may perpetuate a chronic inflammatory state or trigger allograft intolerance syndrome, manifesting as fever, pain, or haematuria.^1^

Managing immunosuppression in these cases is critical but remains a debated topic. The continuation of low-dose immunosuppression can preserve residual kidney function, minimise antibody-mediated rejection, and prevent acute rejection or allograft intolerance syndrome.^1,7^ However, it also increases the risk of infection, malignancy, and cardiovascular complications.^1,3^ Current guidelines are lacking, but decisions often hinge on balancing these risks against potential benefits.

Timing dialysis reinitiation in patients with failed allografts is another critical challenge. Evidence suggests that early dialysis initiation, based on higher eGFR, does not confer survival benefits and may increase mortality risks, possibly reflecting confounding by indication.^1,3^ Late dialysis initiation, guided by clinical symptoms rather than strict eGFR thresholds, is generally preferred to optimise outcomes.^1,3^ In cases of severe complications such as liquefaction, dialysis timing should be dictated by the urgency of the patient’s clinical status.

Liquefaction represents an extreme form of allograft failure, likely involving infectious or ischaemic mechanisms. This complication emphasises the importance of monitoring for atypical presentations post-transplantation. Infectious complications remain a leading cause of morbidity and mortality in renal transplant recipients, particularly in the presence of substantial immunosuppression.^1,8^ In this context, timely diagnosis and intervention, including potential nephrectomy, are paramount.

Transplant nephrectomy in failed allograft is often reserved for symptomatic cases, such as persistent infection, chronic pain, or allograft intolerance syndrome.^1,3^ However, nephrectomy carries risks, including sensitisation, which can complicate future transplantation.^1^ The increase in sensitisation post nephrectomy is due to residual donor tissue including a vascular cuff, in the setting of immunosuppression withdrawal or minimisation.^9,10^ In the context of liquefaction, nephrectomy might be unavoidable, serving both therapeutic and diagnostic purposes.

This case underscores the complexity of managing failed renal transplants, particularly in the presence of rare complications like liquefaction. A multidisciplinary approach with individualised patient assessment, vigilant monitoring, judicious immunosuppression, and timely interventions is vital for optimising outcomes. Further research is warranted to elucidate the pathophysiology, refine treatment strategies, and develop evidence-based guidelines for managing extreme allograft complications such as liquefaction.
